# Colonisation Patterns of *Nosema ceranae* in the Azores Archipelago

**DOI:** 10.3390/vetsci9070320

**Published:** 2022-06-25

**Authors:** Ana Rita Lopes, Raquel Martín-Hernández, Mariano Higes, Sara Kafafi Segura, Dora Henriques, Maria Alice Pinto

**Affiliations:** 1 Centro de Investigação de Montanha, Instituto Politécnico de Bragança, Campus de Santa Apolónia, 5300-253 Bragança, Portugal; ana.lopes@ipb.pt (A.R.L.); dorasmh@ipb.pt (D.H.); 2 Laboratorio de Patología Apícola, IRIAF—Instituto Regional de Investigación y Desarrollo Agroalimentario y Forestal, Centro de Investigación Apícola y Agroambiental (CIAPA), Consejería de Agricultura de la Junta de Comunidades de Castilla-La Mancha, Camino de San Martín, 19180 Marchamalo, Spain; rmhernandez@jccm.es (R.M.-H.); mhiges@jccm.es (M.H.); 3 Instituto de Recursos Humanos para la Ciencia y la Tecnología (INCRECYT-FSE/EC-ESF), Fundación Parque Científico y Tecnológico de Castilla—La Mancha, 02006 Albacete, Spain; 4 Zoología y Antropología Física, Facultad de Ciencias Biológicas, Universidad Complutense de Madrid, 28014 Madrid, Spain; skafafisegura@gmail.com

**Keywords:** *Apis mellifera*, honey bee, *Nosema apis*, real-time qPCR, prevalence, infection levels, *Varroa destructor*, *Vairimorpha*

## Abstract

**Simple Summary:**

*Nosema ceranae* is an emergent honey bee pathogen that has now invaded most of the world. However, geographically-isolated places that are free of this pathogen may still exist, and the Azores may be one of them. Here, we used molecular tools to see whether *N. ceranae* has entered the Azores and how far it has colonised the archipelago. In 2014/2015 we sampled 474 colonies from eight islands, and in 2020 we re-sampled 91 colonies from four islands. Our results showed that *N. ceranae* was present on all islands but Santa Maria and Flores. In the 2014/2015 sampling, Pico, the island of *Varroa destructor* entry in the Azores, showed the greatest prevalence. Resampling in 2020 revealed that *N. ceranae* built-up on Terceira and São Jorge. Our findings suggest that *N. ceranae* colonised the archipelago recently, and it spread across the other islands. Santa Maria is also free of *V. destructor*, making it one of the remaining areas in the world where bees are naive to both stressors. This study will help the veterinary authority establish biosecurity rules for the movement of bees and hive products among islands to maintain the *N. ceranae*-free status of Santa Maria and Flores.

**Abstract:**

*Nosema ceranae* is a highly prevalent pathogen of *Apis mellifera*, which is distributed worldwide. However, there may still exist isolated areas that remain free of *N. ceranae*. Herein, we used molecular tools to survey the Azores to detect *N. ceranae* and unravel its colonisation patterns. To that end, we sampled 474 colonies from eight islands in 2014/2015 and 91 from four islands in 2020. The findings revealed that *N. ceranae* was not only present but also the dominant species in the Azores. In 2014/2015, *N. apis* was rare and *N. ceranae* prevalence varied between 2.7% in São Jorge and 50.7% in Pico. In 2020, *N. ceranae* prevalence increased significantly (*p* < 0.001) in Terceira and São Jorge also showing higher infection levels. The spatiotemporal patterns suggest that *N. ceranae* colonised the archipelago recently, and it rapidly spread across other islands, where at least two independent introductions might have occurred. Flores and Santa Maria have escaped the *N. ceranae* invasion, and it is remarkable that Santa Maria is also free of *Varroa destructor*, which makes it one of the last places in Europe where the honey bee remains naive to these two major biotic stressors.

## 1. Introduction

Western honey bees (*Apis mellifera* Linnaeus; Hymenoptera: Apidae) play a crucial role in the maintenance of ecosystems’ biodiversity through their pollination services. Yet, in recent decades, honey bee populations have declined worldwide [1,2], compromising not only food security but also current and future income for farmers and beekeepers [3]. Various factors, such as climate change, pesticide exposure, and malnutrition, together with predators (e.g., *Vespa velutina* Lepeletier; Hymenoptera: Vespidae), and pathogens (e.g., *Varroa destructor* Anderson and Trueman (Mesostigmata: Varroidae), viruses, bacteria, and fungi) have been identified as interacting contributors to honey bee colony losses [1,2,4,5]. Within fungi, the microsporidia of the former genus *Nosema* play an important role in honey bee health [6,7]. A recent phylogenetic study reclassified the genus *Nosema* as *Vairimorpha* [8]. However, here we will keep the name *Nosema*, for the sake of consistency with the existing literature. 

*Nosema* spp. (Microsporidia: Nosematidae) are obligate intracellular parasites of the ventricular host cells, which are infectious to honey bees and other Hymenoptera [9,10]. In honey bees, the following three species have been identified: *Nosema apis* Zander [11], *Nosema ceranae* Fries et al. [12] and, most recently, *Nosema neumanni* Chemurot et al. [13]. Until the early 2000s, nosemosis in *A. mellifera* was thought to be caused only by *N. apis,* while *N. ceranae* was thought to be species-specific to *Apis cerana* Fabricius. However, this view changed when Higes et al. [14] and Huang et al. [15] detected *N. ceranae* for the first time in *A. mellifera*, in Spain and Taiwan, respectively. Since the host shift episode, *N. ceranae* has become the most prevalent microsporidia in *A. mellifera* populations across the globe [16,17,18,19], whereas *N. neumanni* has only been described in Uganda. Of the three species, *N. ceranae* is the one that raises more concern and has been pointed out as an important culprit of colony demise, at least in countries with warmer climates [6,20,21,22,23,24]. *N. ceranae* can harm honey bees in many different ways, namely, the following: (i) by decreasing lifespan and nursing ability [25,26], (ii) by inducing precocious foraging [26,27,28,29], (iii) by affecting olfactory learning, memory [30], and flight [31]. All these effects can cause decreases in colony size, honey production, and brood-rearing capacity, eventually leading to the collapse of the entire colony [6,32,33].

The islands offer an interesting stage for studying the colonisation dynamics of invasive pathogens such as *N. ceranae,* due to the geographic isolation of the host populations. Nonetheless, there are only a few reports on *N. ceranae’s* prevalence on islands, and these include the Canadian Prince Edward Island and Newfoundland [34], Caribbean Sea islands such as Cuba [35] and Dominica [36], and Pacific Ocean islands such as New Zealand and Norfolk [37,38]. In Europe, surveys of *N. ceranae* have been reported for Ireland, Great Britain [39,40], Isle of Man [41], Tuscan archipelago [42], and the Canary Islands [43]. The presence of *N. ceranae* was confirmed on all islands with a prevalence ranging from 7% (Isle of Man) to 100% (Norfolk), whereas *N. apis* was detected only in Dominica, New Zealand, Prince Edward Island, Newfoundland, Ireland, and Great Britain, with a prevalence ranging from 21.9% (Prince Edward Island and Newfoundland) to 82.3% (Dominica).

The Azores is a Portuguese archipelago in the Macaronesia region, located over 1400 km west of Lisbon. The archipelago is composed of nine islands, all harbouring honey bee colonies that are predominantly near the coastline. The first introduction of honey bees dates back to the XVI century, when Portuguese settlers brought in colonies to the largest island of São Miguel [44], probably from northern Portugal [45]. The last introduction occurred in 2015 when colonies from the island of Terceira were taken to the smallest island of Corvo [46]. There are now nearly 8000 managed hives in the Azores [47], with the islands of São Miguel, Terceira, and Pico harbouring the largest number of apiaries (76%) and the most active beekeeping [48]. 

Due to geographical isolation, the Azores were free of one of the most important honey bee stressors, the invasive ectoparasitic mite *V. destructor,* until 2000 [45]. The mite hitchhiked in the queen’s parcels illegally imported to Pico in 2000 and to the island of Flores in 2001 [45]. Eight years after the first introduction, the mite was spotted on the island of Faial, which is only 8.3 km apart from Pico. Contrary to *V. destructor*, which introduction history is well-known, whether *N. ceranae* reached and spread throughout the archipelago was uncertain. 

Since 2008, *Nosema* spp. has been annually surveyed in the Azores by the regional veterinary authority. According to the annual reports, the only islands that are negative to the microsporidia are the islands of Santa Maria and Flores, together with the most recently colonised island of Corvo, while the island of São Jorge became positive in 2018. [48]. However, since spore detection was based on morphological methods under light microscopy, the question remains as to which *Nosema* species underlies the detection. This is because the spores of *N. apis* and *N. ceranae* are very similar, and there is no data on field symptoms that could help discriminate between the two species. It was supposed that nosemosis was caused by *N. apis,* the species associated with *A. mellifera* before the recent worldwide spread of *N. ceranae* [16,18,19,49,50], as the geographical isolation and the early biosecurity measures, imposed by the veterinary authority after arrival of the *V. destructor* to Pico (Dr. Paula Vieira, *Direção Regional da Agricultura e Desenvolvimento Rural dos Açores*, pers. comm.), could be able to deter the new honey bee pathogen from entering the Azores. However, if *N. ceranae* succeeded in reaching the Azores, its transmission among honey bee populations that are isolated on each island would be difficult, allowing unique conditions for studying an emergent pathogen colonising a pristine territory. While *V. destructor* and *N. ceranae* are serious pests for a colony [32,51,52], when they are simultaneously present, it is possible that their impact is further aggravated by a summative or synergistic interaction. Whether this interaction exists is unclear, although some studies have found a positive correlation between the two parasites [53,54,55,56,57]. In the event that *N. ceranae* is detected, then the Azores provide a unique stage for addressing this issue because there are islands where the mite is present and islands that are mite free. Hereby, the objectives of this study were to (i) determine whether *N. ceranae* is present in the Azores; (ii) infer its prevalence and levels of infection throughout the archipelago; (iii) search for any association between *N. ceranae* and *V. destructor*; finally, (iii) explore possible colonisation scenarios of this emergent pathogen.

## 2. Materials and Methods

### 2.1. Survey and Sampling Collection

**Figure 1:**
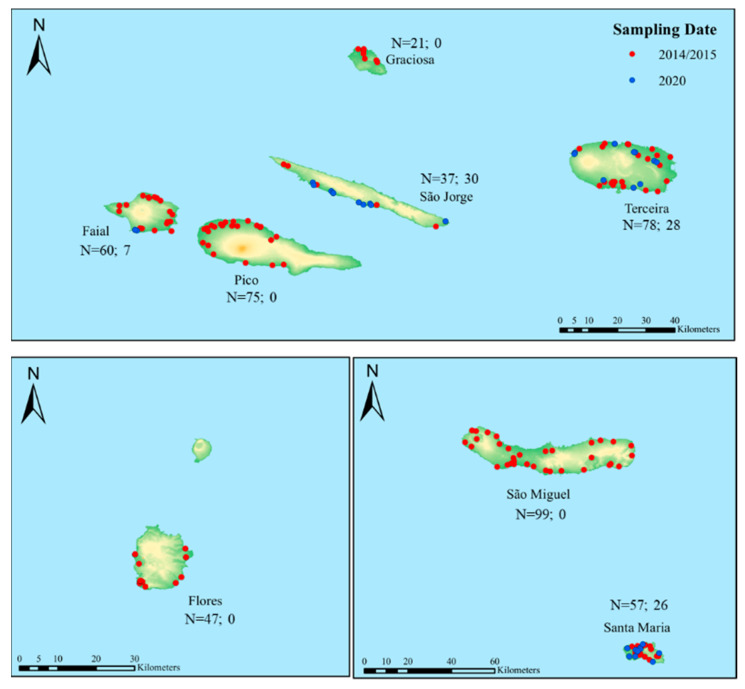
Geographic distribution of the apiaries sampled in 2014/2015 (red dots) and 2020 (blue dots) across the Azores. N is the number of colonies sampled in 2014/2015 (**left**) and 2020 (**right**).

The number of colonies to be sampled was calculated in relation to the number of apiaries registered in 2013 with an expected prevalence of *Nosema* species of 15%, a precision rate of 10% and a confidence level of 95%. The number of samples was subsequently distributed in proportion to the number of apiaries on each island in which colonies were selected randomly (Table S1). In this cross-sectional study, 474 colonies (representing a total of 156 apiaries) were sampled from eight Azorean islands in July and August of 2014 and 2015 (Figure 1 and Table 1). While most apiaries were represented by three colonies, there were a few apiaries that were represented by either two or four colonies (Table S2). Over 150 workers were collected from the outside frame of brood nest of the 474 colonies to appropriate card boxes and then shipped alive to *Centro de Investigação de Montanha* (CIMO; latitude 41°47′53.19″ N, longitude 6°45′56.89″ W).

Given the rise of *Nosema* spp. on São Jorge in 2018, as detected by the veterinary authority, in 2020 we decided to re-sample this island and the following three additional islands for comparison purposes: Santa Maria (island negative to *Nosema* spp., according to the morphological reports), Faial (island positive to *V. destructor*) and Terceira (island negative to *V. destructor* but positive to *Nosema* spp., according to the morphological reports). A total of 91 colonies, representing 34 apiaries, were sampled between July and August of 2020 using the same protocol as in 2014/2015 (Figure 1 and Table 1). The 565 samples were stored at −80 °C until molecular analyses.

### 2.2. Nosema *spp.* Extraction and Detection for the Prevalence Study

Total DNA was extracted from a pool of 120 workers for each of the 474 samples (collected in 2014/2015), following the protocol described in Martín-Hernández et al. [58] with minor modifications. Briefly, 120 workers were added to a double bag strainer (BA6040, Seward, Worthing, UK) with 18 mL of 50% AL Buffer (Qiagen^®^, Hilden, Germany) and crushed in Stomacher 80 (Stomacher 80-Microbiomaster^®^, Seward, Worthing, UK) for two minutes at low speed, followed by adding 9 mL of 50% AL buffer and one more cycle of homogenization (60 s). Subsequently, those macerates were centrifuged at 3000 rpm for 10 min, and the pellet was resuspended in 3 mL of MilliQ water. To improve DNA yield, the mechanical lysis of the spores was applied by adding 400 µL of resuspended pellet to a 96-well plate (Qiagen^®^, Hilden, DE) containing glass beads (2 mm diameter, Sigma), which was shaken in a TissueLyser machine (Qiagen^®^, Hilden, DE) for three minutes. A negative control with no honey bee sample was set for every 20 samples and processed in parallel. Finally, 150 µL of macerate was used for DNA extraction following the BS96 DNA Tissue extraction in a BioSprint workstation (Qiagen^®^, Hilden, DE). The DNA extracts were frozen at −20 °C until the next analysis.

*N. ceranae*, *N. apis* and an internal control of *A. mellifera* DNA (COI gene) were simultaneously screened by using a multiplex-PCR developed by Martín-Hernández et al. [58]. PCR primers are shown in Table S3 whereas the thermal profile and reactions set up are detailed in Martín-Hernández et al. [58]. Nontemplate (NTC) and positive controls were included in each run. PCR products were analysed by capillary electrophoresis in the QIAxcel advanced apparatus using a QIAxcel DNA High Resolution Kit (Qiagen^®^, Hilden, DE).

### 2.3. N. ceranae Extraction and Load Determination

Since the previous DNA extracts of the 2014/2015 sampling period were accidentally lost, DNA was isolated *de novo* from a pool of 30 remaining workers and from all the samples collected in 2020, for real-time qPCR purposes. Fifteen per cent of the colonies were excluded for further analysis due to the insufficient number of workers to pool, reducing the sample size of the 2014/2015 collection to 403 (Table S2). These samples were used to determine the *N. ceranae* load and to compare the prevalence between both periods (2014/2015 and 2020). Briefly, the 30 workers were placed in a double bag strainer (BA6040, Seward, Worthing, UK) and added 6 mL of cool DEPC water (E476, VWR, Pennsylvania, US). Subsequently, the homogenization step was carried out using the MixWell Lab Blender (Alliance Bio Expertise^®^, Guipry-Messac, FR) for the following two cycles: one of 60 s followed by another 30 s, with 30 s pause between each cycle. DNA was extracted from the tissue homogenates by using the standard protocol for animal tissue from NucleoSpin Tissue commercial kit (Macherey-Nagel^TM^, Düren, Germany), with minor modifications. To that end, 50 µL of the homogenates and 180 µL of T1 buffer were added to 2 mL tubes containing two zirconia beads (3 mm, Specanalítica Lda., Carcavelos, PT) to proceed with the mechanical tissue disruption using Precellys (Bertin Instruments, Montigny-le-Bretonneux, FR) with the following protocol: 6200 rpm; 5 s; 3 times. Subsequently, the NucleoSpin Tissue protocol extraction was followed without any modification. The yield and DNA quality were verified through spectrophotometry (Lvis chip, SpectroStar Nano, BMG Labtech, Ortenberg, Germany) and DNA extracts were normalized to 10 ng/µL.

Our approach to quantifying *N. ceranae* load per colony combined the primers described by Martín-Hernández et al. [58] and the SYBR^®^ Green chemistry (primers shown in Table S3). Real-time qPCR assays were performed in the QuantStudio 5 apparatus (Applied Biosystems^®^, Masschusetts, USA). qPCR reactions were carried out in 10 µL total volume (two replicates per sample), containing 10 ng of DNA, 5 µL of 2× iTaq Universal SYBR^®^ Green Supermix (Biorad^®^, California, USA), 300 nM of each primer (Table S3) and 2 µL of DEPC water. The thermal conditions were set according to the SYBR^®^ Green manufacturer’s instructions. Positive controls used to establish the standard curves were made by concentrating known positive samples for *N. ceranae* through PCR. PCR products were quantified using the LVis Chip (SpectroStar Nano, BMG Labtech, Ortenberg, Germany) apparatus. Subsequently, PCR products were used to prepare ten-fold serial dilutions with concentrations (7 points in duplicate) ranging from 1.75 × 10^−2^ to 1.75 × 10^−8^ ng/µL (Figure S1). Melting curve analysis was conducted to detect primer dimers and/or unspecific amplicons. All samples amplifying before the last point of the standard curve, with an amplification plot showing an exponential increase and a melting profile matching the melting temperature of the positive controls, were classified as *N. ceranae* positive. In addition, the RPL8 gene of *A. mellifera* was analysed in those samples to check whether DNA extraction was successful ([59]; see primers in Table S3). *N. ceranae* loads were used to establish the following three infection classes: low for loads < 10^−8^ ng/µL, medium for loads between 10^−5^ ng/µL and 10^−8^ ng/µL and, high for loads ≥ 10^−5^ ng/µL.

### 2.4. Statistical Analysis

To evaluate *N. ceranae* prevalence differences between sampling periods and its association with *V. destructor*, the Chi-square test (χ^2^) was applied. Whenever the sample size of one of the variables was smaller than 5 counts, one of the Chi-square test assumptions was violated, and the Fisher’s exact test was applied. The strength of the association between *N. ceranae* prevalence and *V. destructor* (presence/absence at the island level) was assessed with Cramer’s V measurement (φ_c_). 

Since the qPCR data did not meet the assumptions of parametric tests, differences in *N. ceranae* loads among islands were assessed using the Kruskal–Wallis or Mann–Whitney tests. All statistical analyses and graphical representations were carried out in R studio software (version 4.0.2) [60] and the level of significance was set at 95% (α = 0.05).

## 3. Results

### 3.1. Quality Control

All samples analysed with the multiplex PCR method amplified the internal control of *A. mellifera* DNA (COI gene), confirming DNA integrity. Furthermore, none of the DNA extraction controls or non-template controls (NTCs) were amplified, suggesting no cross-contamination during sample processing and analysis. Regarding real-time qPCR, all samples validated DNA integrity by amplifying the *A. mellifera* RPL8 gene, and all NTCs showed no amplification, indicating that there was no cross-contamination throughout the study. The amplification efficiency of *N. ceranae* was 97.4% (Figure S1). Altogether, these quality control results indicate that the data analysed below is of good quality.

### 3.2. Detection and Prevalence of Nosema *spp.* across the Azores

**Figure 2:**
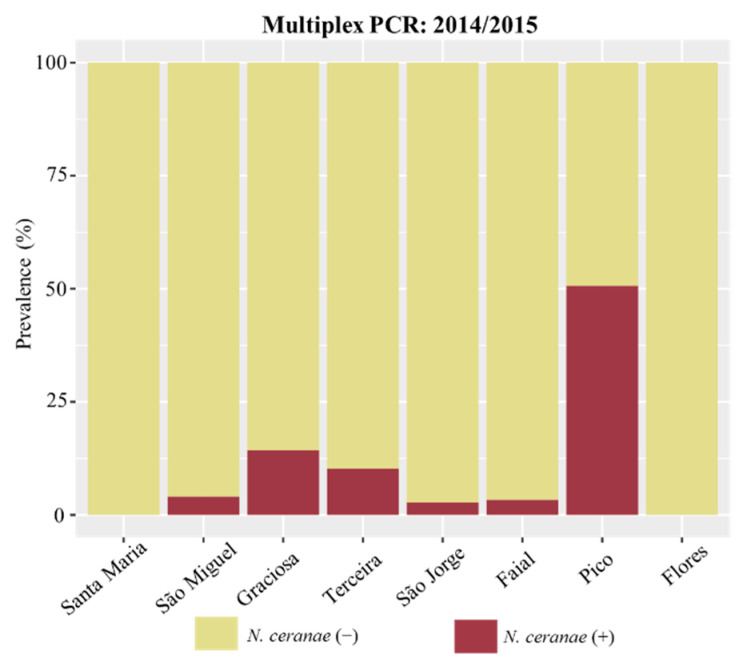
*N. ceranae* prevalence result of multiplex approach in 2014/2015.

The prevalence of *Nosema* infection in the Azores was inferred from the multiplex-PCR output for the samples from the 2014/2015 period, as allowed by the sampling design. A total of 56 (11.8%), 9 (1.9%), and 6 (1.3%), out of 474 colonies, tested positive for *N. ceranae*, *N. apis,* and co-infection, respectively, using the multiplex PCR approach (Table 2 and Figure 2). *N. ceranae* showed the highest prevalence on Pico (50.7%), followed by Graciosa and Terceira, with 14.3% and 10.3%, respectively. On São Miguel, Faial and São Jorge, *N. ceranae* prevalence was ≤4.0% and Santa Maria and Flores did not have any positive colony. *N. apis* was detected in five islands (Flores, Graciosa, São Jorge, São Miguel, and Terceira), in general, with a lower prevalence than *N. ceranae,* ranging from 1.3 to 5.1% (Table 2). The co-infection was even rarer, with one positive colony in São Jorge (2.7%), one positive colony in Terceira (1.3%), and four positive colonies in São Miguel (4.0%).

**Figure 3:**
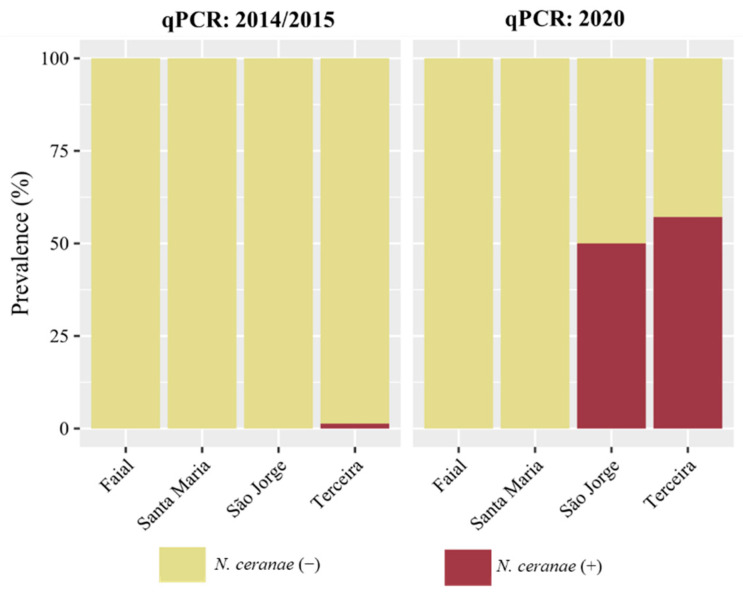
*N. ceranae* prevalence result of qPCR in 2014/2015 compared with 2020.

To assess whether the infection changed between 2014/2015 and 2020, samples were processed for both periods using the same qPCR conditions. The overall prevalence for the first sampling period showed that 34 (8.4%) of the 403 screened colonies were positive for *N. ceranae* (Table 2 and Figure 3). *N. ceranae* could be detected only on Pico (43.7%), Graciosa (5.6%), Terceira (1.4%), and São Miguel (1.1%). On Faial, the two colonies that tested positive for *N. ceranae* by the multiplex PCR approach were negative by qPCR. The single positive colony of São Jorge was not re-analysed because there were not enough workers for pooling. In the 2020 sampling, the highest number of *N. ceranae* positive colonies was recorded on Terceira (57.1%), closely followed by São Jorge (50%). No infection of *N. ceranae* was identified on Faial and Santa Maria. Notably, the months of July and August of 2020 were considerably dryer than those of 2014 and 2015 (Table 1), which may have influenced the build-up of the infection observed on these two islands. 

While the overall prevalence patterns are consistent between the qualitative PCR (multiplex) and the quantitative approach (qPCR), a reanalysis of the 2014/2015 dataset by qPCR, using a lower number of pooled workers (30 instead of 120), led to the detection of a lower number of positives, indicating that false negatives may arise from small pool sizes. This is expected when the proportion of infected individuals in the colony is low, requiring a higher pool size for detecting infected colonies [61]. 

Statistical analysis of *N. ceranae* prevalence data, obtained from qPCR, revealed a highly significant difference (*p* < 0.001; Chi-square test) between sampling periods (Figure 3), which is explained by the dramatic rise in positive colonies observed in 2020 for Terceira and São Jorge (*p* = 0.001 for both islands; Fisher’s exact test).

### 3.3. N. ceranae Loads across the Azores

**Figure 4:**
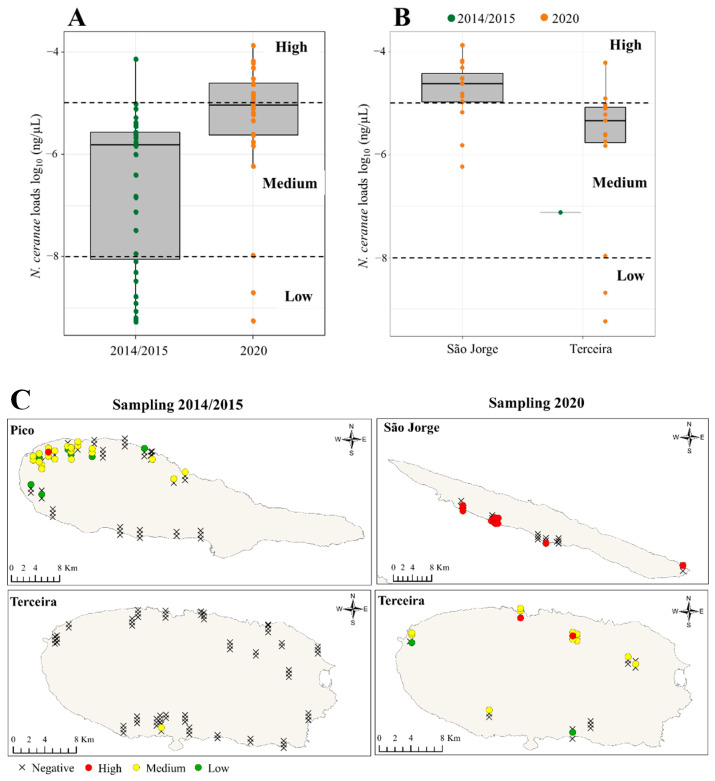
*N. ceranae* infection loads by sampling period and island. (**A**) Boxplot showing the distribution of the positive samples in 2014/2015 (green dots) and 2020 (orange dots); (**B**) Boxplot comparing the N. ceranae loads between the island sampled in 2014/2015 (green dots) and 2020 (orange dots); (**C**) Geographic distribution of the samples with infection levels classified as high (red dots); medium (yellow dots) and low infection (green dots). Negative samples are denoted by a cross mark.

*N. ceranae* loads were statistically different (*p* < 0.0001; Mann–Whitney test) between the 2014/2015 and 2020 sampling periods, with the latter showing the highest median loads (Figure 4A). In the 2014/2015 sampling period, the level of *N. ceranae* infection in positive colonies ranged from 5.44 × 10^−10^ ± 5.49 × 10^−11^ to 7.34 × 10^−5^ ± 1.69 × 10^−6^ ng/µL (Figure 4A). Despite this wide range, the levels of *N. ceranae* infection across islands were not significantly different from each other (*p* = 0.175; Kruskal–Wallis test), since most of the positive colonies in this sampling period originated from Pico. The other three positive islands (Graciosa, Terceira, and São Miguel), as detected by the qPCR approach (Table 2), only had one positive colony each and the infection level was generally low (<1.04 × 10^−8^ ng/µL). In the 2020 sampling period, the two islands (São Jorge and Terceira) with *N. ceranae* positive colonies had a broad range of infection levels (from 5.74 × 10^−10^ ± 6.72 × 10^−11^ to 1.35 × 10^−4^ ± 1.52 × 10^−5^ ng/µL; Figure 4B). São Jorge recorded the highest median (2.40 × 10^−4^ ± 9.10 × 10^−6^ ng/µL) of *N. ceranae* loads, being statistically different from Terceira (*p* = 0.001; Mann–Whitney test). 

When categorising the infection level of *N. ceranae* according to the qPCR loads in the 2014/2015 sampling period, most colonies exhibited a medium infection (23; 75%), one colony exhibited a severe infection (3.1%), and seven colonies exhibited a low infection (21.9%). Notably, the majority of the medium-infected colonies originated from Pico, and they were located on the north-western part of the island (Figure 4C). In the 2020 sampling period, 14 (45.2%) positive colonies were categorised as having a high infection, 15 (48.4%) a medium infection, and 2 (6.4%) a low infection. The most highly infected colonies originated from São Jorge (Figure 4B).

### 3.4. N. ceranae and V. destructor Associations

**Figure 5:**
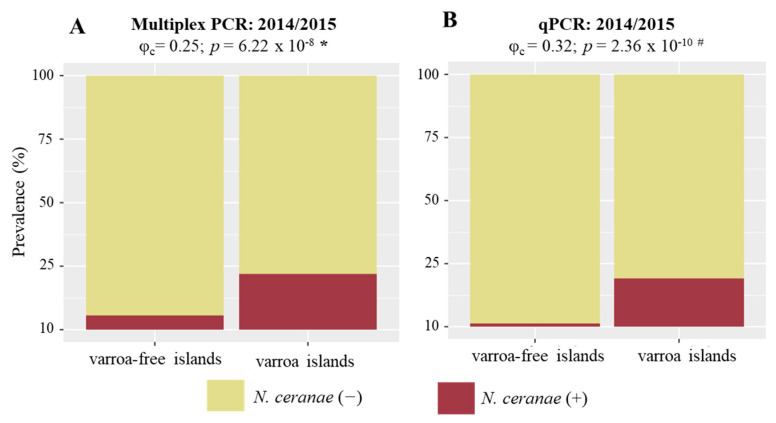
*N. ceranae* prevalence for the islands with and without *V. destructor* for the 2014/2015 sampling period. (**A**) multiplex PCR prevalence between islands with and without *V. destructor*; (**B**) real-time qPCR prevalence between islands with and without *V. destructor*. The strength of the association between the two parasites was evaluated by Cramer’s V (<0.1–0.3: weak; 0.3–0.5: moderate; >0.5: strong) and the significance of the association by the Chi-square Test (*) or Fisher’s exact test (^#^).

The association between *N. ceranae* prevalence and *V. destructor* was tested using the multiplex PCR and the qPCR datasets separately. A statistically significant positive association was found in the 2014/2015 sampling period (φ_c_ = 0.25; *p* < 0.001, Chi-square test) (Figure 5A). This finding was corroborated by the qPCR qualitative data (φ_c_ = 0.32; *p* < 0.001, Fisher’s exact test), despite the lower number of tested samples (Figure 5B). In contrast, the prevalence of *N. apis* (φ_c_ = 0.08; *p* = 0.163, Fisher’s exact test) or co-infection (φ_c_ = 0.09; *p* = 0.087, Fisher’s exact test) did not reveal any relationship with *V. destructor* presence. Since only one (Faial) of the sampled islands from 2020 has *V. destructor*, the association analysis could not be performed.

## 4. Discussion

This study documents for the first time the presence of *N. ceranae* in the Azores, further extending its distributional range in the world (reviewed by Klee et al. [16] and Grupe and Quandt [49]) and specifically in Macaronesia, where it had only been reported for the Canaries [43]. *Nosema* infection has occurred in the Azores at least since 2008, according to the first spore identification by microscopical analysis produced by the local veterinary authority [46]. Whether the early cases were due to *N. apis* or *N. ceranae* is unknown. Moreover, unknown is the year and exact location of the first arrival of *N. ceranae* to the archipelago, contrasting with the known putative *N. ceranae*-free status of the following two of its islands: Santa Maria and Flores. Remarkably, despite the widespread distribution of *N. ceranae* in the Azores, samples from these two islands were all negative in both the 2014/2015 and 2020 sampling periods. These molecular results, together with the morphological results reported by the Azorean veterinary authority for several years (from 2008 to 2021; *Direção Regional da Agricultura e Desenvolvimento Rural dos Açores* [46,48]), strongly suggest that Santa Maria and Flores are still free of this harmful pathogen. 

In the 2014/2015 sampling, *N. ceranae* prevalence was below 10.3% for six of the eight surveyed islands, with Santa Maria and Flores exhibiting values of 0%. However, in the 2020 sampling, while *N. ceranae* went undetected on Santa Maria and Faial, the epidemiological situation aggravated considerably in São Jorge and Terceira, as suggested by over 50% of samples testing positive in qPCR on both islands. Due to the geographically limited sampling effort in 2020, these results should be interpreted with caution, particularly on Faial where only two apiaries were examined. Nonetheless, the increasing trend observed on São Jorge and Terceira, which is supported by the morphological data [48], cannot be ignored. These findings call for a thorough molecular survey across the entire archipelago so that a more rigorous appraisal of the epidemiological status of the Azorean honey bee populations, and a further confirmation of the *N. ceranae*-free status of Flores and Santa Maria, can be carried out. This is particularly important for Santa Maria as this island is also free of *V. destructor* and harbours the purest Iberian honey bee (*A. m. iberiensis*) population in the Azores [45].

Since the first discovery in colonies sampled in 2005 in Spain [14] and in Taiwan [15], *N. ceranae* has been detected with high prevalence rates in all continents where *A. mellifera* is present, indicating a very high invasive potential [16,49,62]. In the European continent, *N. ceranae* reached 95–98% prevalence in Hungary [63], 84% in the Balkan countries [20], 77.2% in the northern part of Bulgaria [64], 59.7% in Belgium [65], over 63.2% in Italy [23,66], or 58.5% in Spain [67]. In mainland Portugal, the possible origin of the spores introduced in the Azores, *N. ceranae* prevalence was over 60% in a survey conducted in 2012 (report published by *Federação Nacional dos Apicultores de Portugal* [68]). High prevalence rates have also been reported for islands. For instance, *N. ceranae* reached 97.1% prevalence in Cuba [35], 63% in the Dominica Islands [36], or 75% in the Macaronesia archipelago of the Canaries [43]. 

All those regions were in stark contrast with the epidemiological situation of the Azores, where two islands seemed to remain free of *N. ceranae* and five islands exhibited prevalence rates lower than 14.3% in the 2014/2015 sampling. Only Pico, the island where *V. destructor* was first introduced, registered a large proportion of infected colonies, regardless of the molecular approach used (43.7–50.7%). This result suggests that, as for *V. destructor*, Pico might have acted as the first entry point for *N. ceranae* in the Azores. If this was the case, it is possible that *N. ceranae* spores hitchhiked along with *V. destructor* in the queens parcels illegally imported in 2000, as this pathogen has been shown to be infecting *A. mellifera* since at least 1995 in the USA [17] and 1998 in Europe [69]. Alternatively, Pico did not act as the entry point in the Azores and *N. ceranae* could have been introduced later to this island through contaminated hive products [70,71,72], originating from the varroa-free islands, mainland or elsewhere, and *V. destructor* aided infection development by lowering the immune defences of the honey bees [73,74,75]. If the first hypothesis is true, then the stringent restrictions on the circulation of honey bees and hive products from the varroa-invaded islands onto the varroa-free islands, imposed early by the veterinary authority (Dr. Paula Vieira, pers. comm.), imply that at least one additional independent introduction of *N. ceranae* occurred on São Miguel, São Jorge, Terceira, or Graciosa. On Flores, the other illegal queen import was seemingly free of *N. ceranae*, as suggested by the negative results obtained from either molecular or morphological methods. 

The introduction of *N. ceranae* on the varroa-free islands likely occurred via commercial hive products, as the importation of queens or packaged honey bees has been restricted for over 22 years. Honey, pollen, royal jelly, and wax foundation can all contain viable spores and thus operate as transmission vehicles of *N. ceranae* through trading [70,71,72]. Among these, the wax foundation is the more probable original source of introduction in the varroa-free islands because there is a high demand for this hive product in the Azores and sterilisation of wax imports has only been compulsory since 2010 (Dr. Paula Vieira, pers. comm.). Regardless of the places and means of *N. ceranae’s* entrance, the spatial and temporal prevalence patterns suggest that *N. ceranae* arrived recently in the Azores, and it is invading the archipelago at a fast pace. Since its entry, it is behaving as an emerging pathogen, as evidenced by the rising prevalence observed in some islands.

In addition to prevalence, *N. ceranae* infection loads were determined for all positive samples identified by real-time qPCR. In 2014/2015, the three single positive samples, originating from varroa-free islands, had low (Graciosa and São Miguel) and medium (Terceira) infection intensities. On Pico, there were samples with low as well as high infection intensities, but the great majority (75%) exhibited medium intensity, and these were mostly located in the northwest. This part of the island has a higher concentration of apiaries, which might have facilitated the transmission of *N. ceranae* as the spores are considered bioaerosols that can be carried in the air and deposited in natural environments, including flowers [76]. Other ways to spread the infection could be through sharing food or water sources, or the effects of drifting or robbing infected colonies. In the 2020 sampling, the two islands (Terceira and São Jorge) that showed high prevalence also showed high loads, particularly São Jorge. Strikingly, in the previous sampling, only one sample was identified as positive on this island, and now, in addition to the high prevalence (50.0%), most samples displayed infection levels classified as high. The infection loads, together with the temporal prevalence patterns, suggest a recent establishment of *N. ceranae* on these two varroa-free islands, and now the pathogen is rapidly spreading, showing the typical behaviour of an emergent pathogen, with an increase in the prevalence and in the level of infection. Therefore, despite the positive association between *V. destructor* and *N. ceranae* prevalence detected in an earlier stage of the invasion (2014/2015), and also reported in other studies [53,54,55,56,57], these findings suggest that the mite is not a mandatory condition for a successful invasion of the pathogen.

Once *N. ceranae* is introduced into a pristine territory, several beekeeper-independent factors, such as pesticide exposure, quality of pollen forage, and climate, can influence the establishment of the pathogen and the build-up of the infection [77,78,79,80,81,82]. Yet, new introductions into geographically isolated places such as the Azores are dependent on humans for transportation of propagules. In the case of *N. ceranae*, long-distance transmission is facilitated by the trade of infected honey bees or contaminated hive products [70,71]. There are only two islands, Flores and Santa Maria (and perhaps Corvo), that remain free of *N. ceranae* (according to the veterinary report for 2021 [48]), and this status can only be perpetuated if biosecurity restrictions are capable of continuing to prohibit any importation attempt of honey bees and decontamination of imported hive products used in beekeeping (e.g., wax) is assured.

## 5. Conclusions

This is the first molecular survey of *Nosema* spp. carried out in the Azores and consequently the first detection of *N. ceranae* in this Macaronesia region. *Nosema* spp. was identified on some of the islands in the first morphological report released by the local veterinary authority in 2008. Whether these early cases were due to *N. apis* or *N. ceranae* is unknown. However, there is a chance that *N. ceranae* was already present in 2008, at least on Pico, as suggested by the high prevalence rate and infection loads found on this island in the 2014/2015 sampling. The spatial and temporal patterns are compatible with a recent colonisation hypothesis, after *N. ceranae* has been introduced in the Azores, likely multiple times. Flores and Santa Maria have so far seemed to avoid *N. ceranae* invasion, a situation that can be sustained if beekeepers comply with biosecurity regulations. While Flores has *V. destructor*, Santa Maria is free of this parasite. Therefore, Santa Maria is one of the last places in Europe, and perhaps in the world, where honey bees remain naive to two of the major honey bee biotic stressors, making this island unique for beekeeping activity.

## Figures and Tables

**Table 1:** Sample sizes and climate data for the 2014/2015 and 2020 sampling periods. Islands with *V. destructor* are represented in bold.

Island	2014/2015				2020			
	Colonies	Apiaries	Temperature (°C) *	Rainfall (mm) *	Colonies	Apiaries	Temperature (°C) **	Rainfall (mm) **
Santa Maria	57	19	22.4/23.1	54.7/30.9	26	12	21.4/23.4	15.0/36.7
São Miguel	99	33	21.6/22.5	49.7/58.3	-	-	20.8/22.8	7.1/19.5
Graciosa	21	7	22.0/22.5	64.3/91.6	-	-	***	***
Terceira	78	26	22.5/23.2	52.8/35.8	28	10	21.6/23.1	9.1/33.8
São Jorge	37	13	21.5/21.7	67.1/45.3	30	10	21.2/22.8	18.0/33.8
**Faial**	60	20	22.1/22.8	117.5/63.3	7	2	21.6/22.8	16.5/87.0
**Pico**	75	25	22.6/23.3	127.6/43.1	-	-	22.5/23.4	11.8/86.5
**Flores**	47	13	22.3/22.8	116.6/165.2	-	-	22.3/23.0	12.2/131.3
Total	474	156			91	34		

* Monthly average July (2014/2015)/August (2014/2015); ** Monthly average July/August 2020; *** Unavailable information; Data obtained from *Instituto Português do Mar e da Atmosfera*.

**Table 2:** Prevalence of *N. ceranae*; *N. apis;* co-infection by sampling year and molecular method. The number of positive samples and sample sizes are shown within parenthesis for each island. Islands with *V. destructor* are represented in bold.

Island	*N. ceranae*			*N. apis*	Co-Infection
	2014–2015		2020	2014–2015	2014–2015
	Multiplex PCR	qPCR	qPCR	Multiplex PCR	Multiplex PCR
Santa Maria	nd (0/57)	nd (0/45)	nd (0/26)	nd (0/57)	nd (0/57)
São Miguel	4.0% (4/99)	1.1% (1/91)	-	5.1% (5/99)	4.0% (4/99)
Graciosa	14.3% (3/21)	5.6% (1/18)	-	4.8% (1/21)	nd (0/21)
Terceira	10.3% (8/78)	1.4% (1/74)	57.1% (16/28)	1.3% (1/78)	1.3% (1/78)
São Jorge	2.7% (1/37)	nd (0/13)	50.0% (15/30)	2.7% (1/37)	2.7% (1/37)
**Faial**	3.3% (2/60)	nd (0/54)	nd (0/7)	nd (0/60)	nd (0/60)
**Pico**	50.7% (38/75)	43.7% (31/71)	-	nd (0/75)	nd (0/75)
**Flores**	nd (0/47)	nd (0/37)	-	2.1% (1/47)	nd (0/47)

nd = not detected.

## Data Availability

The data analyzed for the study are available from the corresponding author on reasonable request.

## Supplementary Materials

The following supporting information can be downloaded at: https://www.mdpi.com/article/10.3390/vetsci9070320/s1; Figure S1: Real-time qPCR standard curves for *N. ceranae*; Table S1: Distribution of registered apiaries and colonies per island in 2013; Table S2: Metadata of sampled apiaries; Table S3: Primers used for molecular detection of *N. ceranae*; *N. apis* and internal controls for *Apis mellifera*.
