# Supplementary material for: Colonisation Patterns of Nosema ceranae in the Azores Archipelago
Source: Vet Sci. 2022 Jun 25;9(7):320. doi: 10.3390/vetsci9070320 (PMC9323992; doi:10.3390/vetsci9070320)
Supplement: Supplementary file 1 [file vetsci-09-00320-s001.zip › vetsci-1774635-supplementary.pdf]

Supplementary Materials

Table S1. Distribution of registered apiaries and colonies per island in 2013; and the corresponding theoretical sample sizes determined for an expected prevalence of *Nosema* species of 15%; a precision rate of 10% and a confidence level of 95%. Islands with *V. destructor* are represented in bold.

Island	Registered apiaries	Registered colonies	Number of apiaries to sample	Number of colonies to sample
Santa Maria	45	231	19	57
São Miguel	272	1802	30	90
Terceira	123	954	26	78
Graciosa	10	167	7	21
São Jorge	36	161	17	51
**Pico**	92	1020	25	75
**Faial**	49	286	20	60
**Flores**	29	133	15	45
Total	656	4754	159	477

Table S2. Metadata of sampled apiaries.

Island	Sampling year	Apiary ID	multiplex PCR	qPCR	Coordinate X	Coordinate Y
			Number of colonies sampled	Number of colonies sampled		
Faial	2015	F1	3	2	−28.736	38.526
Faial	2020	F1	0	4	−28.736	38.526
Faial	2015	F10	3	3	−28.764	38.604
Faial	2015	F11	3	3	−28.693	38.625
Faial	2015	F12	3	3	−28.626	38.552
Faial	2015	F13	3	2	−28.623	38.522
Faial	2015	F14	3	3	−28.785	38.602
Faial	2015	F15	3	2	−28.694	38.628
Faial	2020	F16	0	3	−28.731	38.523
Faial	2015	F17	3	2	−28.628	38.583
Faial	2015	F18	3	3	−28.676	38.524
Faial	2015	F19	3	3	−28.657	38.617
Faial	2015	F2	3	3	−28.669	38.625
Faial	2015	F20	3	3	−28.637	38.546
Faial	2015	F21	3	3	−28.676	38.629
Faial	2015	F3	3	3	−28.786	38.584
Faial	2015	F4	3	3	−28.711	38.528
Faial	2015	F5	3	2	−28.715	38.530
Faial	2015	F6	3	3	−28.635	38.551
Faial	2015	F7	3	2	−28.712	38.632
Faial	2015	F8	3	3	−28.721	38.531
Faial	2015	F9	3	3	−28.620	38.573
Flores	2015	FL1	4	4	−31.244	39.390
Flores	2015	FL10	3	3	−31.249	39.393
Flores	2015	FL11	4	3	−31.247	39.392
Flores	2015	FL12	5	4	−31.262	39.455
Flores	2015	FL13	4	0	−31.252	39.432

Flores	2015	FL2	4	2	−31.153	39.401
Flores	2015	FL3	4	4	−31.142	39.448
Flores	2015	FL4	3	2	−31.144	39.468
Flores	2015	FL5	1	1	−31.252	39.433
Flores	2015	FL6	2	2	−31.166	39.387
Flores	2015	FL7	3	3	−31.251	39.387
Flores	2015	FL8	3	3	−31.238	39.379
Flores	2015	FL9	6	6	−31.250	39.392
Graciosa	2014	G1	3	2	−28.040	39.091
Graciosa	2014	G2	3	3	−28.022	39.076
Graciosa	2014	G3	3	2	−28.019	39.060
Graciosa	2014	G4	3	3	−28.022	39.084
Graciosa	2014	G5	3	2	−27.982	39.055
Graciosa	2014	G6	3	3	−27.979	39.050
Graciosa	2014	G7	3	3	−28.022	39.091
Pico	2015	P1	3	3	−28.508	38.521
Pico	2015	P10	3	3	−28.498	38.540
Pico	2015	P11	3	3	−28.433	38.539
Pico	2015	P12	3	2	−28.521	38.528
Pico	2015	P13	3	3	−28.417	38.538
Pico	2015	P14	3	3	−28.469	38.544
Pico	2015	P15	3	3	−28.362	38.418
Pico	2015	P16	3	2	−28.307	38.415
Pico	2015	P17	3	3	−28.272	38.417
Pico	2015	P18	3	3	−28.508	38.477
Pico	2015	P19	3	3	−28.512	38.532
Pico	2015	P2	3	3	−28.464	38.539
Pico	2015	P20	3	2	−28.430	38.552
Pico	2015	P21	3	3	−28.384	38.554
Pico	2015	P22	3	2	−28.312	38.494
Pico	2015	P23	3	3	−28.295	38.504
Pico	2015	P24	3	3	−28.454	38.549
Pico	2015	P25	3	3	−28.344	38.535
Pico	2015	P3	3	3	−28.464	38.541
Pico	2015	P4	3	3	−28.489	38.536
Pico	2015	P5	3	3	−28.391	38.423
Pico	2015	P6	3	3	−28.524	38.485
Pico	2015	P7	3	3	−28.355	38.540
Pico	2015	P8	3	3	−28.345	38.535
Pico	2015	P9	3	3	−28.491	38.449
São Jorge	2015	SJ1	3	2	−28.169	38.667
São Jorge	2020	SJ10	0	3	−28.022	38.604
São Jorge	2014	SJ11	3	1	−28.272	38.731
São Jorge	2020	SJ12	0	3	−28.002	38.607
São Jorge	2015	SJ13	3	1	−27.796	38.537
São Jorge	2014	SJ14	3	1	−27.766	38.553
São Jorge	2020	SJ14	0	3	−27.766	38.553
São Jorge	2020	SJ15	0	3	−27.998	38.604
São Jorge	2014	SJ16	3	0	−28.152	38.661
São Jorge	2015	SJ17	3	0	−28.223	38.708
São Jorge	2015	SJ18	3	0	−28.133	38.658
São Jorge	2020	SJ2	0	3	−28.178	38.665

São Jorge	2015	SJ20	2	0	−28.013	38.603
São Jorge	2015	SJ21	3	0	−28.177	38.670
São Jorge	2020	SJ3	0	3	−28.181	38.674
São Jorge	2015	SJ4	3	1	−28.258	38.725
São Jorge	2014	SJ5	3	2	−27.981	38.603
São Jorge	2020	SJ6	0	3	−28.123	38.647
São Jorge	2020	SJ7	0	3	−28.117	38.641
São Jorge	2015	SJ7	3	2	−28.117	38.641
São Jorge	2020	SJ8	0	3	−28.118	38.642
São Jorge	2014	SJ9	3	3	−28.037	38.612
São Jorge	2020	SJ9	0	3	−28.037	38.612
São Miguel	2014	SM1	3	3	−25.708	37.742
São Miguel	2014	SM10	3	2	−25.583	37.754
São Miguel	2014	SM11	3	3	−25.202	37.743
São Miguel	2014	SM12	3	3	−25.639	37.779
São Miguel	2014	SM13	3	3	−25.347	37.730
São Miguel	2014	SM14	3	3	−25.840	37.844
São Miguel	2014	SM15	3	3	−25.317	37.780
São Miguel	2014	SM16	3	3	−25.795	37.891
São Miguel	2014	SM17	3	2	−25.237	37.754
São Miguel	2014	SM18	3	3	−25.149	37.789
São Miguel	2014	SM19	3	3	−25.441	37.727
São Miguel	2014	SM2	3	3	−25.637	37.767
São Miguel	2014	SM20	3	3	−25.506	37.727
São Miguel	2014	SM21	3	2	−25.480	37.810
São Miguel	2014	SM22	3	1	−25.316	37.843
São Miguel	2014	SM23	3	2	−25.698	37.837
São Miguel	2014	SM24	3	3	−25.243	37.750
São Miguel	2014	SM25	3	3	−25.650	37.756
São Miguel	2014	SM26	3	3	−25.709	37.868
São Miguel	2014	SM27	3	2	−25.793	37.857
São Miguel	2014	SM28	3	3	−25.556	37.743
São Miguel	2014	SM29	3	2	−25.665	37.751
São Miguel	2014	SM3	3	3	−25.280	37.852
São Miguel	2014	SM30	3	3	−25.637	37.754
São Miguel	2014	SM31	3	3	−25.660	37.818
São Miguel	2014	SM32	3	3	−25.152	37.831
São Miguel	2014	SM33	3	3	−25.812	37.894
São Miguel	2014	SM4	3	3	−25.225	37.846
São Miguel	2014	SM5	3	3	−25.613	37.791
São Miguel	2014	SM6	3	3	−25.487	37.723
São Miguel	2014	SM7	3	3	−25.507	37.807
São Miguel	2014	SM8	3	3	−25.814	37.827
São Miguel	2014	SM9	3	3	−25.746	37.886
Santa Maria	2015	STM1	3	1	−25.114	36.995
Santa Maria	2020	STM10	0	1	−25.103	37.007
Santa Maria	2015	STM11	3	3	−25.095	37.004
Santa Maria	2015	STM12	3	3	−25.097	37.003
Santa Maria	2015	STM13	3	1	−25.122	36.966
Santa Maria	2015	STM14	3	3	−25.073	36.999
Santa Maria	2020	STM15	0	1	−25.037	36.970
Santa Maria	2015	STM16	3	2	−25.047	36.957

Santa Maria	2015	STM17	3	3	−25.076	36.943
Santa Maria	2020	STM18	0	3	−25.133	36.984
Santa Maria	2015	STM19	3	1	−25.135	36.979
Santa Maria	2015	STM2	3	3	−25.149	36.997
Santa Maria	2020	STM20	0	2	−25.062	36.935
Santa Maria	2015	STM21	3	2	−25.126	37.000
Santa Maria	2015	STM22	3	1	−25.129	36.970
Santa Maria	2020	STM23	0	2	−25.135	36.956
Santa Maria	2020	STM24	0	2	−25.159	36.956
Santa Maria	2020	STM25	0	2	−25.124	36.979
Santa Maria	2015	STM26	3	3	−25.114	36.968
Santa Maria	2020	STM26	0	3	−25.114	36.968
Santa Maria	2020	STM27	0	3	−25.127	36.976
Santa Maria	2015	STM28	3	3	−25.069	36.987
Santa Maria	2015	STM29	3	3	−25.039	36.957
Santa Maria	2015	STM3	3	2	−25.096	36.955
Santa Maria	2015	STM30	3	3	−25.121	36.989
Santa Maria	2020	STM4	0	3	−25.168	36.991
Santa Maria	2015	STM5	3	3	−25.111	36.994
Santa Maria	2015	STM6	3	3	−25.116	36.964
Santa Maria	2015	STM7	3	2	−25.136	36.968
Santa Maria	2020	STM8	0	3	−25.115	36.993
Santa Maria	2020	STM9	0	1	−25.114	36.988
Terceira	2020	T1	0	3	−27.106	38.737
Terceira	2014	T10	3	3	−27.285	38.663
Terceira	2014	T11	3	2	−27.237	38.676
Terceira	2014	T12	3	3	−27.269	38.796
Terceira	2014	T13	3	3	−27.074	38.678
Terceira	2020	T14	0	3	−27.273	38.681
Terceira	2020	T15	0	3	−27.115	38.742
Terceira	2020	T16	0	1	−27.363	38.763
Terceira	2014	T17	3	2	−27.197	38.794
Terceira	2014	T18	3	3	−27.210	38.662
Terceira	2014	T2	2	2	−27.105	38.758
Terceira	2014	T20	3	3	−27.361	38.767
Terceira	2020	T20	0	3	−27.361	38.767
Terceira	2014	T21	3	3	−27.364	38.765
Terceira	2014	T22	3	3	−27.276	38.786
Terceira	2014	T23	3	3	−27.247	38.672
Terceira	2014	T24	3	3	−27.096	38.727
Terceira	2014	T25	3	3	−27.270	38.674
Terceira	2014	T26	3	3	−27.164	38.759
Terceira	2020	T27	0	3	−27.157	38.668
Terceira	2014	T28	3	2	−27.348	38.780
Terceira	2020	T29	0	3	−27.178	38.769
Terceira	2014	T3	3	3	−27.194	38.793
Terceira	2014	T30	3	3	−27.242	38.665
Terceira	2014	T31	3	3	−27.140	38.649
Terceira	2014	T32	3	3	−27.178	38.657
Terceira	2020	T32	0	3	−27.178	38.657
Terceira	2020	T33	0	3	−27.173	38.768
Terceira	2014	T34	3	3	−27.215	38.676

Terceira	2014	T4	3	3	−27.064	38.754
Terceira	2014	T5	3	2	−27.134	38.748
Terceira	2014	T6	3	3	−27.237	38.795
Terceira	2020	T6	0	3	−27.237	38.795
Terceira	2014	T7	3	3	−27.243	38.675
Terceira	2014	T8	4	4	−27.120	38.778
Terceira	2014	T9	3	3	−27.102	38.646

Table S3. Primers used for molecular detection of *N. ceranae*; *N. apis* and internal controls for *Apis mellifera*.

Primer name		Sequence	Locus	Specificity	Fragment length	Molecular method	Source
218MITOC	forward	5′-CGCCGACGATGTGATATGAAAATATTAA-3′	16S rRNA	*N. ceranae*	218-219 bp	multiplex PCR/qPCR	[58;82]
	reverse	5′-CCCGGTCATTCTCAAACAAAAAACCG-3'					
321APIS	forward	5′-GGGGGCATGTCTTTGACGTACTATGTA-3′	16S rRNA	*N. apis*	321 bp	multiplex PCR	
	reverse	5′-GGGGGGCGTTTAAAATGTGAAACAAVTATG-3′					
COI	forward	5′-GGGTCCAAGACCAGGAACTGGAT-3′	mtDNA citochrome oxidase subunit I	*A. mellifera*	119 bp	multiplex PCR	[58]
	reverse	5′-GCGCGGAAATTCCTGATATATGAAGAGAAAA-3′					
RPL8	forward	5′-TGGATGTTCAACAGGGTTCATA-3′	RPL8	*A. mellifera*	121 bp	qPCR	[59]
	reverse	5′-CTGGTGGTGGACGTATTGATAA-3′					

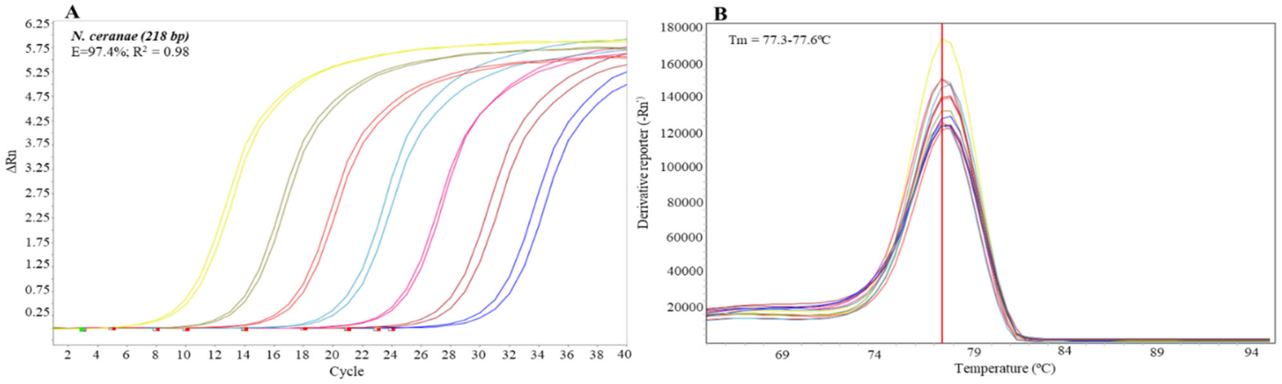

Figure S1. Real-time qPCR standard curves for *N. ceranae*. Amplification curves and melting profile (A; B) of ten-fold serial dilutions (7 points in duplicate).
